# Effectiveness of Comprehensive Health Education Combining Lifestyle Education and Hot Spa Bathing for Male White-Collar Employees: A Randomized Controlled Trial with 1-Year Follow-Up

**DOI:** 10.2188/jea.JE20081020

**Published:** 2009-09-05

**Authors:** Hiroharu Kamioka, Yosikazu Nakamura, Shinpei Okada, Jun Kitayuguchi, Masamitsu Kamada, Takuya Honda, Yuzuru Matsui, Yoshiteru Mutoh

**Affiliations:** 1 Faculty of Regional Environment Science, Tokyo University of Agriculture, Tokyo, Japan; 2 Department of Public Health, Jichi Medical University, Shimotsuke, Tochigi, Japan; 3 Physical Education and Medicine Research Foundation, Tomi, Nagano, Japan; 4 Physical Education and Medicine Research Center Unnan, Unnan, Shimane, Japan; 5 Department of Physical and Health Education, Graduate School of Education, The University of Tokyo, Tokyo, Japan; 6 Department of Orthopedics, Unnan Hospital, Unnan, Shimane, Japan

**Keywords:** male, white-collar-employees, randomized controlled trial, lifestyle education, hot spa, and exercise

## Abstract

**Background:**

Physical activity is known to prevent obesity and metabolic syndrome in middle-aged and elderly people; however, the effectiveness of a comprehensive health education program for male white-collar employees is uncertain.

**Methods:**

Forty-three men volunteered to participate in this study and were randomly assigned into 2 groups. The intervention group participated in a 2-hour program comprising comprehensive health education and hot spa bathing, offered once every 2 weeks, in addition to individualized programs once a week, for 24 weeks. The control group received only general health guidance. We compared their lifestyle characteristics and physical and mental health criteria at baseline, immediately after the intervention, and 1 year after the end of the intervention.

**Results:**

Rates of adherence to individualized programs were 60.0 ± 27.2% and 30.5 ± 29.6% at the end of the intervention and at 1 year after the end of the intervention, respectively. Significant (*P* < 0.05) interaction of criteria was observed for cluster of differentiation 4+ (CD4+) cells and the ratio of cluster of differentiation 4+ to 8+ (CD4/8) cells, which were used to represent the participants' immunological function. We divided the intervention group into 2 subgroups on the basis of their attendance. Among the resulting 3 groups, significant interaction of criteria was observed for CD4+ and CD4/8 cells. In addition, the high attendance group had the highest CD4+ count and CD4/8 ratio.

**Conclusions:**

Participants who attended classes and/or performed the supplementary individualized programs tended to maintain their immunological function and to experience a decrease in body fat percentage. However, few effects were noted in participants with poor adherence, even in the intervention group.

## INTRODUCTION

The prevalences of obesity and metabolic syndrome are increasing in many industrialized countries.^1^ In Japan, the prevalence of metabolic syndrome, as determined using Japanese criteria, was 18.4% and 5.8% for men and women, respectively.^2^ Among the indicators of metabolic syndrome, high blood pressure was most frequently observed, followed by dyslipidemia; high fasting plasma glucose was least frequent among both sexes.

The health benefits of physical activity for middle-aged and elderly people are well documented, and an increase in physical activity is effective in preventing coronary heart disease, stroke, diabetes, obesity, and hypertension, and for improving quality of life and mental health.^3^^–^^10^ An increasing number of people enjoy bathing in hot spas, and more hot spa facilities are being built throughout Japan.^11^ Spa bathing (balneotherapy, or spa therapy) is a popular alternative medical treatment, and is a very popular treatment for arthritis in many European countries, as well as in Israel and Japan.^12^^–^^14^ Hot spas exert a thermal action, hydraulic pressure, a chemical action, and a general conditioning action,^15^ all of which are known to affect humans favorably. Several studies suggest that the warmth and buoyancy of spa water block nociception by acting on thermal receptors and mechanoreceptors and by enhancing blood flow, which is thought to help in dissipating algogenic chemicals and in possibly facilitating muscle relaxation.^16^^,^^17^ The hydrostatic effect may relieve pain by reducing peripheral edema^18^ and by dampening sympathetic nervous activity.^19^ Balneotherapy may also have potential for augmenting immunological function (suppressor T cells, natural killer cell activity, B cells, cluster of differentiation 4+ cells, etc.) and may relieve stress.^20^ We hypothesized that in Japan, where hot spa bathing is a part of daily life and custom, hot spa bathing (including bathing at home), when combined with a health education program focused on enhancing conventional physical and health activities, should improve health. However, no randomized controlled trials (RCTs) have been performed to determine the effects of such a comprehensive health education program for male white-collar employees.

In the present study, we instructed male white-collar employees on exercise, diet, and daily life activities once every 2 weeks for 24 weeks. They were also encouraged to take hot spa baths so that we could evaluate the health effects of spa bathing. Serum lipid levels and immune function were the main outcome measures and were monitored for 1 year.

## METHODS

### Participants and randomization

**Figure 1.**
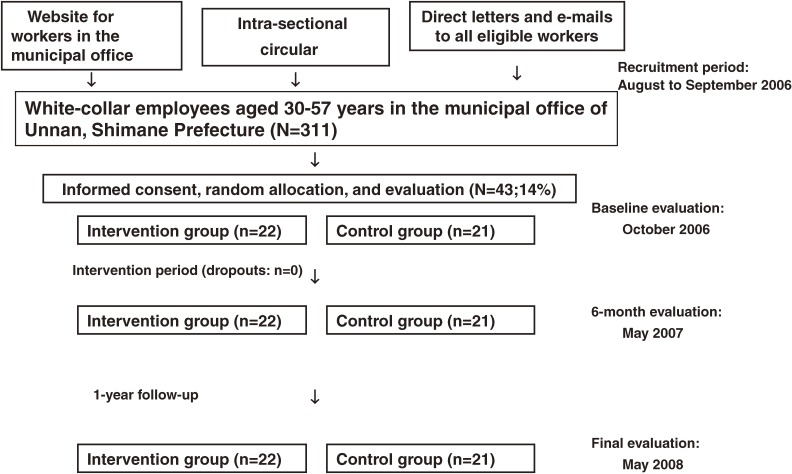
Study flow diagram

Figure 1 shows the study flow diagram. This study was announced between August and September 2006 on a website used exclusively by municipal office personnel in Unnan, Japan. The eligibility criteria were male sex, age from 30 to 57 years, and no contraindications for exercise or spa bathing. Workers were informed of the study by means of the website in the municipal office, intrasectional circular, and direct letters and e-mails to all eligible people. Of 311 male white-collar employees between the ages of 30 and 57 years, 43 volunteered to participate in this study. After explanatory meetings were held on September 25–26, all 43 volunteers (14% participation rate) agreed to be included in this study, regardless of whether they were assigned to the control group or the intervention group (no blinding for participants). Twenty-two and 21 volunteers were randomly assigned by lottery to the intervention and control groups, respectively. We first randomized the list of volunteer names using a common bingo lottery device, after assigning unique numbers to the volunteers in the list in the order of their application. We then assigned 22 signed sticks (intervention group) and 21 unsigned sticks (control group), which were drawn from sealed boxes, to the numbers for the volunteers. The lottery operation was performed by a third party, ie, a person other than authors, and the person in charge of intervention and evaluation. Concealment was confirmed after completion of the assignments.

### Research design

In this RCT we compared comprehensive health education to limited health education. No dropouts were reported during the intervention period; therefore, all analyses were intention-to-treat (ITT). In addition, the intervention group was divided into high (*n* = 10) and low (*n* = 12) adherence groups, and subgroup analysis was then performed between the 3 groups, ie, the high and low adherence groups and the control group. The trial procedure, analysis, and description were reported according to the CONSORT statement^21^ and the CLEAR NPT checklist (a checklist to evaluate reports of nonpharmacological trials).^22^

### Intervention and setting

A 2-hour program encompassing comprehensive health education with hot spa bathing was offered once every 2 weeks for 24 weeks (Table 1). Guidance on lifestyle, physical exercise, and diet consisted of lectures (comprehensive health education) and various forms of physical exercise, as shown in Table 1. Each session required approximately 60 minutes. The lectures and exercise sessions were delivered by physicians, dieticians, academics, public health nurses, and exercise instructors with more than 20, 5, 10, 15, and 5 years, respectively, of clinical or pedagogical experience.

**Table 1.** Protocol for lifestyle education, exercise, and bathing^a^

Session	Date^b^	Instructor	Main program (contents)
1	Oct. 30–31	public health nurse	A lecture on project significance and setting goals
2	Nov.13–14	orthopedist	A lecture on preventing backache and stiff shoulders
3	Nov.27–28	exercise instructor	Light exercise: stretching
4	Dec.11–12	internist	A lecture on preventing lifestyle-related diseases
5	Dec.25–26	exercise instructor	Light exercise: stretching and walking
6	Jan.15–16	psychiatrist	A lecture on releasing mental stress
7	Jan.29–30	exercise instructor	Light exercise: recreation (1)
8	Feb.13–14	exercise instructor	Light exercise: recreation (2)
9	Feb.26–27	academic expert	A lecture on modifying behavior for health
10	Mar.12–13	dietician	A lecture on appropriate eating for office workers
11	Mar.27–28	exercise instructor	Light exercise: recreation (3)
12	Apr.9–10	public health nurse	A lecture on self-management of health

^a^All lectures and exercises were 60 min, and were followed by spa bathing in earthy gypsum salt springs for 30–45 min.

^b^To increase participation, the lectures and exercise sessions were held on more than 1 day.

The participants took half-baths (up to the chest) in an open-air bath (earthy gypsum salt spring; bath temperature, 40 °C). The duration of the bath was approximately 20 minutes (2 baths of 10 minutes each). The total time for the bathing process was approximately 60 minutes, which included 40 minutes for changing clothes, washing the body, and resting (drinking beverages). The public multipurpose hall, located at a distance of 200 meters from the municipal hall, and the spa facility, which was immediately adjacent to the multipurpose hall, were used as settings for the intervention. All participants were able to visit the spa facility, which was within a 10-minute drive from their home or workplace.

In addition to the intervention listed in Table 1, the volunteers also participated in an individualized program once a week. This program focused on targets set by the participants themselves, and included physical activities in addition to spa bathing (for example, a 30-minute walk before returning home, in addition to their daily activities).

Based on studies showing that written reports circulated after the completion of an intervention resulted in higher rates of persistent effects,^23^^,^^24^ we sent copies of a newsletter to the participants once every 3 months (4 times) during the 1-year observation period. These digests (1 sheet of A4-sized paper) contained a review of recommended methods for eating, sleeping, exercising, and bathing.

Participants in the control group received general health guidance on a single occasion, in addition to instructions on stretching and dietary guidance. They were also encouraged to continue with their usual daily activities.

### Instructions on daily living

Guidance on daily living during the intervention emphasized increased physical activity, such as walking and using stairs instead of an escalator or elevator. Regarding dietary guidance, a leaflet was distributed that indicated the suggested level of energy intake for the program. Daily bathing at home or a spa was recommended at a suitable water temperature (40°–41 °C) in a half-bath (water level at the chest while seating). In addition, participants were asked to perform their individualized programs as frequently as possible, although these instructions were delivered in a nonsupervisory manner, so as to give priority to the participant’s free will, work conditions, and family situation.

### Outcome and process measurements

The baseline assessment was performed in October 2006, the interim assessment was performed immediately after completion of the intervention in May 2007, and the final assessment was done after a 1-year observation period, in May 2008. Serum HDL cholesterol (HDL-C) was the main outcome measure, and hemoglobin A1c (HbA1c) level and the ratio of cluster of differentiation 4+ and 8+ (CD4/8) were defined as secondary outcomes. Other outcome measures included body measurements, physical strength, blood profile, and mental state. The body measurements were height, weight, body mass index (BMI), waist circumference, hip circumference, and body fat percentage, as measured by the impedance method (TBF-102, Tanita Corp., Japan). The blood profile comprised serum glucose, total cholesterol (T-C), triglyceride (TG), LDL cholesterol (LDL-C), free fatty acid (FFA), GOT, GPT, γ-GTP, cholinesterase (ChE), natural killer cell activity (NK), T cell percentage, B cell percentage, and uric acid (UA). These were examined between 9 AM and 11 AM, after a minimum 12-hour fast.

Measures of strength were grip strength, abdominal strength, back strength (Sensor EG-230, Sakai Corp., Japan), and anteflexion. Aerobic capacity was measured on a bicycle ergometer (Ergociser EC-1600, Cateye Corp., Japan) by using a ramp test with a continuous increase in load, starting from an initial load automatically programmed for sex, age, and weight. Physical Work Capacity (PWCHRmax) was calculated as 100% of HRmax, estimated from sex and age. The Profile of Mood States (POMS)^25^ was used to assess mental state. Participants were asked, while in a quiet and private room, to describe frankly their mood states during the past week.

Process measurements were obtained by using a questionnaire. The lifestyle items were taken from the Japan Arteriosclerosis Longitudinal Study Physical Activity Questionnaire (JALSPAQ)^26^ and included questions on moderate exercise, along with questions on behavior patterns. The number of active modifications of lifestyle was assessed by questionnaire items on the following behaviors: regular breakfast, number of sleep hours, snacking frequency, drinking status, amount of alcohol consumed, smoking status, daily stress, awareness of physical activities, practice of physical activities, spa-bathing times, and bathing times.

All measurements of the body and physical strength were performed by skilled evaluators with more than 5 years of experience. All the measurements of abdominal and back strength were performed by a single evaluator, after sufficient training, because it was considered likely that the evaluator's skill would influence the evaluation. The evaluator and the interventionist were not the same person; neither was blinded to the participants’ group assignment. Collection and examination of blood were outsourced to the Unnan Hospital; both the phlebotomist and the examiner were blinded to the participants’ group assignment.

Classroom attendance rates were regularly confirmed by reviewing an attendance book, and staff confirmed the attendance of participants at each session. Attendance rates were calculated by dividing the number of attendees by the number of sessions. The participants were interviewed about their adherence (percentage of compliance during the previous period) to the individualized program at the end of the intervention and at the final evaluation. The rates of adherence represent the percentage of program compliance during the intervention period and at 1 year after the end of the intervention.

Participants were informed of the study plan, both verbally and in writing, before their written consent was obtained. The information clearly indicated that they were allowed to quit the study at any time, and included an explanation of all the possible disadvantages of participating in the study. To avoid the appearance of unfairness, participants in the control group received a single session of general health guidance, which included instruction on stretching and dietary guidance. In addition, they received several leaflets on improving fitness, as well as coupons for the spa facility and the gym.

The methodology of this study was approved by the Ethics Board of Tokyo University of Agriculture. The present study was registered as ID 000000607 by the University Hospital Medical Information Network Clinical Trials Registry (UMIN-CTR) in Japan.

### Statistical analysis

Because HDL-C was the primary outcome measure and CD4/8 and HbA1c were secondary outcomes of interest, power calculations were performed for the primary outcome. We used data from the National Nutrition Survey in Japan 2004^27^ on people aged 30 to 59 years to determine the sample size. The standard deviation of the mean was approximately 15 mg/dL in males, and the significant difference level between groups in the current study was 15 mg/dL. Statistical power was set so that the probability of type 1 error (α) was 5% and the probability of type 2 error (β) was 20%. The required sample size was calculated to be 16 or more participants in each arm.

A 2-sample *t* test (Welch test) was used to compare continuous variables between groups. Fisher's exact probability test, the Mann-Whitney test, and the Kruskal-Wallis test were used for analysis of discrete variables. Repeated measures of variance (ANOVA) was used to investigate differences in changes between groups (2 groups × 3 times on ITT analysis; 3 groups × 3 times on subgroup analysis). Variables not filling Mauchly's test of sphericity were analyzed after Greenhouse-Geisser correction. Differences within and among groups were judged to be significant when the significance level was 5% or lower. SPSS 14.0J for Windows was used for statistical analysis.

## RESULTS

Table 2 shows the salient medical history of the participants. No significant differences were found between the 2 groups in age, internal diseases, or orthopedic diseases. The attendance rate for the intervention course was 56.2 ± 24.2% (mean ± SD). Rates of adherence to the individualized programs were 60.0 ± 27.2% and 30.5 ± 29.6% during the intervention period and at 1 year after the intervention, respectively.

**Table 2.** Clinical characteristics of participants

	Intervention Group	Control Group
*n*	22	21
Age (yrs)^a^	41.1 ± 7.5	46.3 ± 7.0
Medical history (Internal medicine)		
​ Diabetes	1 (4.5%)	0 (0%)
​ Dyslipidemia	1 (4.5%)	0 (0%)
​ Hyperuricemia	1 (4.5%)	0 (0%)
​ Hyperparathyroidism	0 (0%)	1 (4.8%)
​ Aortic stenosis	0 (0%)	1 (4.8%)
Medical history (Orthopedics)		
​ Knee Osteoarthrosis	0 (0%)	1 (4.8%)
​ Lumbar spine Osteoarthrosis	0 (0%)	2 (9.5%)
​ Osteoporosis	1 (4.5%)	0 (0%)

The numbers represent the number of patients (percentage), unless otherwise indicated.

^a^Mean ± SD

Table 3 shows results of the ITT analysis between groups at baseline, at the interim evaluation, and at the final evaluation, 1 year after the end of the intervention. No significant differences in HDL-C were observed between groups. Significant (*P* < 0.05) interaction was observed for the secondary outcome measure CD4/8, which was used to represent participants' immunological function. Related to this result, significant (*P* < 0.05) interaction was found between groups for CD4+ and CD8+. No significant difference in HbA1c was found between groups. There was a tendency for serum glucose and vigor to differ between groups, (*P* = 0.057 and *P* = 0.069, respectively). No significant differences were found for the other variables.

**Table 3.** Time-series comparison of physical status, blood profile, and mental state (mean ± SD) in the intervention and control groups

Outcome Measurements	Intervention group (*n* = 22)			Control group (*n* = 21)				*P*-value
	
	baseline*	6 months later	follow-up	baseline*	6 months later	follow-up		
Body measurements								
​ Weight, kg	70.7 ± 9.7	70.4 ± 10.0	70.2 ± 8.6	64.2 ± 5.8	64.1 ± 6.3	63.6 ± 5.6		0.875
​ Body mass index, kg/m^2^	24.2 ± 2.8	24.1 ± 3.1	24.0 ± 2.6	22.8 ± 2.5	22.7 ± 2.5	22.5 ± 2.3		0.772
​ Waist circumference, cm	85.2 ± 7.7	84.8 ± 7.0	85.3 ± 7.0	80.4 ± 5.4	80.7 ± 6.4	80.4 ± 6.1	#	0.619
​ Hip circumference, cm	93.6 ± 4.9	93.4 ± 4.7	93.5 ± 4.5	89.4 ± 4.9	90.1 ± 3.7	89.9 ± 3.2	#	0.530
​ Body fat percentage, %	20.2 ± 5.2	18.7 ± 4.7	17.8 ± 3.8	17.8 ± 3.8	16.5 ± 3.8	15.5 ± 3.1	#	0.972
Physical strength								
​ Right-hand grip, kg	47.6 ± 7.2	49.8 ± 6.3	48.6 ± 6.5	45.5 ± 6.2	46.5 ± 6.0	46.4 ± 7.5		0.537
​ Left-hand grip, kg	45.6 ± 6.7	45.8 ± 7.3	44.7 ± 6.4	44.0 ± 7.1	43.8 ± 7.7	44.4 ± 7.6		0.338
​ Anteflexion, cm	38.3 ± 7.6	41.6 ± 8.9	43.8 ± 8.3	37.9 ± 9.8	38.4 ± 7.5	41.1 ± 9.3		0.212
​ Maximal physical working capacity, W	193.6 ± 61.9	189.2 ± 50.5	189.3 ± 48.3	180.8 ± 38.5	186.8 ± 28.0	187.2 ± 24.0		0.274
​ Abdominal strength, kgf	25.7 ± 7.4	28.7 ± 5.1	33.3 ± 5.4	21.4 ± 6.7	24.9 ± 5.1	28.4 ± 5.9		0.942
​ Back strength, kgf	26.8 ± 6.2	34.1 ± 3.8	37.9 ± 5.1	24.5 ± 6.6	31.2 ± 5.7	35.1 ± 5.0		0.855
Blood profile								
​ Serum glucose, mg/dL	99.8 ± 26.5	99.0 ± 20.7	96.7 ± 22.5	95.5 ± 9.0	99.7 ± 12.1	99.8 ± 12.3		0.057
​ Hemoglobin A_1c_, %	5.2 ± 0.8	5.1 ± 0.6	4.9 ± 0.7	5.1 ± 0.4	5.0 ± 0.4	4.9 ± 0.4		0.434
​ Lactic acid, mg/dL	9.1 ± 3.5	8.1 ± 3.0	8.5 ± 2.4	8.9 ± 4.3	9.9 ± 4.3	9.3 ± 3.6		0.350
​ Total cholesterol, mg/dL	197.1 ± 27.0	212.6 ± 28.7	211.8 ± 37.8	190.2 ± 14.8	189.2 ± 48.8	201.8 ± 30.3		0.205
​ Triglyceride, mg/dL	125.0 ± 114.5	141.0 ± 128.0	188.4 ± 290.0	105.0 ± 52.7	118.7 ± 58.6	142.2 ± 92.0	#	0.579
​ High-density lipoprotein cholesterol, mg/dL	59.5 ± 17.0	63.7 ± 16.0	60.0 ± 15.7	59.0 ± 15.1	61.8 ± 14.5	59.1 ± 14.5		0.792
​ Low-density lipoprotein cholesterol, mg/dL	112.7 ± 32.3	120.7 ± 28.3	120.7 ± 26.7	110.1 ± 17.9	112.7 ± 24.5	115.7 ± 28.9	#	0.667
​ Free fatty acid, mEq/L	0.45 ± 0.22	0.34 ± 0.16	0.28 ± 0.11	0.44 ± 0.15	0.30 ± 0.10	0.30 ± 0.11		0.806
​ Glutamic oxaloacetic transaminase, IU/L	22.0 ± 5.5	20.5 ± 4.9	26.4 ± 9.9	21.0 ± 4.8	19.2 ± 3.6	22.1 ± 3.7	#	0.125
​ Glutamic pyruvic transaminase, IU/L	27.5 ± 16.7	25.4 ± 13.3	29.5 ± 13.8	20.6 ± 8.8	22.3 ± 7.9	25.0 ± 6.6	#	0.396
​ γ-glutamyl transpeptidase, IU/L	52.5 ± 43.3	46.1 ± 29.1	59.0 ± 54.0	43.8 ± 55.2	40.1 ± 47.5	41.0 ± 43.3	#	0.130
​ Cholinesterase, IU/L	346.8 ± 57.8	351.0 ± 55.3	349.5 ± 54.5	357.4 ± 55.6	363.3 ± 58.4	354.4 ± 59.6		0.589
​ NK cytotoxicity, %	33.6 ± 17.9	36.5 ± 15.2	29.7 ± 11.3	35.2 ± 14.9	41.1 ± 13.9	36.1 ± 12.7		0.395
​ T cell, %	87.7 ± 5.6	88.5 ± 4.4	87.3 ± 4.9	87.3 ± 4.0	87.6 ± 4.0	86.2 ± 4.1	#	0.776
​ B cell, %	5.7 ± 5.4	3.1 ± 1.7	5.6 ± 3.1	4.3 ± 3.3	2.6 ± 1.2	5.3 ± 3.0	#	0.558
​ Cluster of differentiation 4+, %	39.7 ± 8.3	37.8 ± 6.1	37.9 ± 6.8	43.7 ± 8.7	39 ± 9.9	37.7 ± 7.3		0.027
​ Cluster of differentiation 8+, %	35.9 ± 8.7	36.9 ± 8.1	36.2 ± 7.5	33.0 ± 7.2	34.6 ± 6.4	35.8 ± 7.2		0.027
​ Cluster of differentiation 4/8	1.21 ± 0.49	1.09 ± 0.37	1.12 ± 0.41	1.44 ± 0.67	1.20 ± 0.50	1.12 ± 0.45	#	0.042
​ Uric acid, mg/dL	6.3 ± 0.9	6.1 ± 1.3	6.1 ± 1.2	5.9 ± 1.3	5.8 ± 1.1	5.5 ± 1.2		0.350
Mental state								
​ -Tension	48.6 ± 9.0	50.7 ± 9.0	49.9 ± 7.9	45.8 ± 5.2	46.3 ± 8.1	47.1 ± 7.7		0.728
​ -Depression	50.6 ± 7.7	50.1 ± 7.4	50.5 ± 7.6	46.9 ± 6.7	48.2 ± 8.7	47.8 ± 7.3		0.666
​ -Anger	50.0 ± 8.0	50.5 ± 6.6	50.0 ± 7.1	44.4 ± 6.5	46.6 ± 7.9	44.7 ± 5.3		0.724
​ -Vigor	43.2 ± 6.7	47.4 ± 9.1	45.5 ± 7.5	43.7 ± 11.3	43.0 ± 11.7	43.3 ± 10.6		0.069
​ -Fatigue	51.0 ± 10.1	52.0 ± 8.6	50.6 ± 8.2	48.8 ± 8.9	50.6 ± 11.0	48.6 ± 10.4		0.900
​ -Confusion	52.7 ± 10.0	50.7 ± 8.2	52.1 ± 9.2	51.0 ± 6.9	51.0 ± 7.0	52.0 ± 9.1		0.457

#: Variables not fulfilling Mauchly's test of sphericity were analyzed after Greenhouse-Geisser correction.

*: There were no significant differences between the 2 groups at baseline (2-sample* t *test).

Table 4 shows changes in the lifestyle process measures of the intervention and control groups from baseline through to the final examination. Time spent on moderate exercise increased over the study period (*P* = 0.053) in the control group. No significant differences were found in other lifestyle process measures. Regarding adherence during the 1-year observation period in the intervention group, the cut-off value that divided the group into 2 equal parts was 25%. Subgroup analysis was performed using 3 groups: a high adherence group (more than 25%), a low adherence group (less than 25%), and the control group (Table 5). Significant (*P* < 0.05) effects were observed in CD4+ and CD4/8, which were maintained in the high adherence group, but tended to decrease in the low adherence and control groups. A greater decrease in body fat percentage was observed in the high adherence group, but was not significant (*P* < 0.07). Antiflexion also tended to improve in the high adherence group.

**Table 4.** Time-series comparison of lifestyle characteristics of the intervention and control groups

Process Measurements		Intervention group (*n* = 22)			Control group (*n* = 21)			*P*-value	
	
		baseline#	6 months later	follow-up	baseline#	6 month later	follow-up		
Regular breakfast	yes	19 (86%)	18 (82%)	18 (82%)	17 (81%)	18 (86%)	19 (91%)	0.162	0.083
	no	3 (14%)	4 (18%)	4 (18%)	4 (19%)	3 (14%)	2 (10%)		
Number of sleep hours*	hours per day	6.9 ± 0.9	6.9 ± 0.9	7.0 ± 0.9	6.9 ± 0.9	6.7 ± 0.8	6.8 ± 0.7	0.825	
Snacking frequency	Seldom	10 (46%)	8 (36%)	9 (41%)	8 (38%)	6 (29%)	10 (48%)	0.917	0.592
	1–2 times a month	0 (0%)	2 (9%)	2 (9%)	1 (5%)	2 (10%)	1 (5%)		
	1–2 times a week	3 (14%)	3 (14%)	6 (27%)	4 (19%)	3 (14%)	5 (24%)		
	3–4 times a week	6 (27%)	3 (14%)	2 (9%)	2 (10%)	4 (19%)	4 (19%)		
	Almost every day	3 (14%)	6 (27%)	3 (14%)	6 (29%)	6 (29%)	1 (5%)		
Drinking status	Drinker	18 (82%)	20 (91%)	16 (73%)	16 (76%)	16 (76%)	16 (76%)	0.984	0.249
	Former drinker	0 (0%)	0 (0%)	0 (0%)	0 (0%)	0 (0%)	0 (0%)		
	Infrequent drinker	4 (18%)	2 (9%)	6 (27%)	5 (24%)	5 (24%)	5 (24%)		
Amount of alcohol consumed*	Units^#^	1.6 ± 0.8	1.2 ± 0.8	1.2 ± 1.0	1.0 ± 0.8	1.4 ± 1.1	1.1 ± 1.0	0.301	
	(1 unit = 500 mL/day of beer, or equivalent)^#^								
Smoking status	Smoker	8 (36%)	7 (32%)	6 (27%)	8 (38%)	7 (33%)	8 (38%)	0.323	0.083
	Former smoker	6 (27%)	6 (27%)	6 (27%)	3 (14%)	5 (24%)	3 (14%)		
	Nonsmoker	8 (36%)	9 (41%)	10 (46%)	10 (48%)	9 (43%)	10 (48%)		
Daily stress	Very high	5 (23%)	5 (23%)	2 (9%)	2 (10%)	3 (14%)	1 (5%)	0.416	0.866
	High	9 (41%)	8 (36%)	11 (53%)	7 (33%)	7 (33%)	4 (19%)		
	Normal	6 (27%)	6 (27%)	6 (27%)	9 (43%)	8 (38%)	15 (71%)		
	Low	2 (9%)	3 (14%)	3 (14%)	3 (14%)	3 (14%)	1 (5%)		
Middle strength exercise*	min per month	404.5 ± 423.1	334.5 ± 377.5	215.1 ± 293.0	162.1 ± 218.9	223.6 ± 298.2	319.5 ± 456.4	0.053	
Awareness of the need for	Always aware	1 (5%)	3 (14%)	0 (0%)	0 (0%)	2 (10%)	1 (5%)	0.607	0.467
physical activity	Usually aware	10 (46%)	8 (36%)	13 (59%)	11 (52%)	7 (33%)	11 (52%)		
	Sometimes aware	6 (27%)	8 (36%)	7 (32%)	5 (24%)	10 (48%)	7 (33%)		
	Seldom aware	5 (23%)	3 (14%)	2 (9%)	5 (24%)	2 (10%)	2 (10%)		
Practice of	Precontemplation	3 (14%)	1 (5%)	2 (9%)	5 (24%)	3 (14%)	6 (29%)	0.434	0.334
physical activities	Contemplation	6 (27%)	4 (18%)	8 (36%)	4 (19%)	5 (24%)	7 (33%)		
	Preparation	5 (23%)	11 (50%)	6 (27%)	5 (24%)	7 (33%)	4 (19%)		
	Action	4 (18%)	0 (0%)	0 (0%)	2 (10%)	3 (14%)	1 (5%)		
	Maintenance	4 (18%)	6 (27%)	6 (27%)	5 (24%)	3 (14%)	3 (14%)		
Frequency of spa bathing*	times per month	1.5 ± 1.6	2.0 ± 2.4	2.0 ± 2.3	1.0 ± 1.9	1.3 ± 1.9	1.3 ± 2.6	0.910	
Frequency of bathing at home*	times per month	22.9 ± 8.5	24.2 ± 6.6	23.0 ± 8.1	23.9 ± 7.5	23.5 ± 6.6	24.1 ± 6.3	0.552	

*: Continuous variables are shown as mean ± standard deviation and were tested after Greenhouse-Geisser correction.

Categorical variables are shown as frequency (percentage).

The Mann-Whitney test was adapted to test the difference in 6-month change between groups (left *P*-value); it was also adapted to the change from baseline to follow-up (right *P*-value).

#: There were no significant differences between the 2 groups at baseline (2-sample* t *test for continuous variables and Fisher's exact probability test or Mann-Whitney test)

**Table 5.** Subgroup analysis of physical status, blood profile, and mental state (mean ± SD)

Outcome Measurements	High adherence group (*n* = 10)			Low adherence group (*n* = 12)			Control group (*n* = 21)			*P*-value
		
	baseline	6 months later	follow-up	baseline	6 months later	follow-up	baseline	6 months later	follow-up	
Body measurements										
​ Weight, kg	72.9 ± 10.7	73.0 ± 11.7	71.7 ± 9.0	68.8 ± 8.9	68.2 ± 8.2	68.9 ± 8.4	64.2 ± 5.8	64.1 ± 6.3	63.6 ± 5.6	0.188
​ Body mass index, kg/m^2^	24.8 ± 3.3	24.8 ± 3.7	24.4 ± 2.7	23.7 ± 2.4	23.5 ± 2.6	23.7 ± 2.6	22.8 ± 2.5	22.7 ± 2.5	22.5 ± 2.3	0.232
​ Waist circumference, cm	86.0 ± 8.2	85.9 ± 7.4	85.5 ± 7.5	84.5 ± 7.5	83.9 ± 6.8	85.1 ± 6.9	80.4 ± 5.4	80.7 ± 6.4	80.4 ± 6.1	0.664
​ Hip circumference, cm	94.8 ± 4.9	94.9 ± 5.1	94.7 ± 4.4	92.6 ± 4.9	92.3 ± 4.1	92.5 ± 4.6	89.4 ± 4.9	90.1 ± 3.7	89.9 ± 3.2	0.902
​ Body fat percentage, %	22.4 ± 6.6	19.8 ± 5.3	17.8 ± 2.6	18.3 ± 2.7	17.8 ± 4.2	17.7 ± 4.8	17.8 ± 3.8	16.5 ± 3.8	15.5 ± 3.1	0.066
Physical strength										
​ Right-hand grip, kg	50.5 ± 5.2	52.1 ± 6.4	50.9 ± 6.2	45.2 ± 7.9	47.9 ± 5.8	46.7 ± 6.4	45.5 ± 6.2	46.5 ± 6.0	46.4 ± 7.5	0.783
​ Left-hand grip, kg	46.6 ± 7.1	48.2 ± 6.1	47.4 ± 6.1	44.8 ± 6.6	43.8 ± 7.9	42.5 ± 5.9	44.0 ± 7.1	43.8 ± 7.7	44.4 ± 7.6	0.199
​ Anteflexion, cm	39.7 ± 8.1	45.4 ± 8.4	46.6 ± 6.7	37.2 ± 7.2	38.4 ± 8.3	41.3 ± 9.1	37.9 ± 9.8	38.4 ± 7.5	41.1 ± 9.3	0.067
​ Maximal physical working capacity, W	205.3 ± 67.6	201.0 ± 54.5	199.7 ± 49.4	183.9 ± 57.8	179.4 ± 47.0	179.9 ± 47.6	180.8 ± 38.5	186.8 ± 28.0	187.2 ± 24.0	0.611
​ Abdominal strength, kgf	26.9 ± 6.8	29.4 ± 5.2	35.1 ± 4.5	24.6 ± 7.9	28.0 ± 5.1	31.6 ± 5.9	21.4 ± 6.7	24.9 ± 5.1	28.4 ± 5.9	0.875
​ Back strength, kgf	29.6 ± 5.3	33.7 ± 3.8	38.7 ± 3.9	24.5 ± 6.1	34.4 ± 3.9	37.2 ± 6.0	24.5 ± 6.6	31.2 ± 5.7	35.1 ± 5.0	0.285
Blood profile										
​ Serum glucose, mg/dL	97.3 ± 5.8	95.4 ± 6.7	93.3 ± 7.5	101.9 ± 36.1	102.1 ± 27.5	99.5 ± 30.1	95.5 ± 9.0	99.7 ± 12.1	99.8 ± 12.3	0.207
​ Hemoglobin A_1c_, %	5.1 ± 0.4	5.1 ± 0.3	4.9 ± 0.4	5.2 ± 1.0	5.2 ± 0.8	5.0 ± 0.9	5.1 ± 0.4	5.0 ± 0.4	4.9 ± 0.4	0.703
​ Lactic acid, mg/dL	9.0 ± 3.5	8.4 ± 2.9	8.7 ± 2.1	9.1 ± 3.7	7.9 ± 3.2	8.3 ± 2.7	8.9 ± 4.3	9.9 ± 4.3	9.3 ± 3.6	0.640
​ Total cholesterol, mg/dL	212.6 ± 30.0	227.5 ± 26.1	218.3 ± 35.9	184.3 ± 16.1	200.3 ± 25.4	206.4 ± 40.0	190.2 ± 14.8	189.2 ± 48.8	201.8 ± 30.3	0.294
​ Triglyceride, mg/dL	109.6 ± 56.1	127.5 ± 53.4	141.8 ± 97.7	137.8 ± 148.5	152.3 ± 169.2	227.3 ± 386.2	105.0 ± 52.7	118.7 ± 58.6	142.2 ± 92.0	0.629
​ High-density lipoprotein cholesterol, mg/dL	60.2 ± 16.3	63.2 ± 14.9	58.4 ± 14.5	59.0 ± 18.3	64.1 ± 17.5	61.3 ± 17.0	59.0 ± 15.1	61.8 ± 14.5	59.1 ± 14.5	0.520
​ Low-density lipoprotein cholesterol, mg/dL	130.5 ± 29.0	138.9 ± 23.9	130.8 ± 29.2	97.9 ± 27.7	105.6 ± 22.6	112.3 ± 22.2	110.1 ± 17.9	112.7 ± 24.5	115.7 ± 28.9	0.419
​ Free fatty acid, mEq/L	0.4 ± 0.1	0.3 ± 0.2	0.3 ± 0.1	0.5 ± 0.3	0.4 ± 0.2	0.3 ± 0.1	0.4 ± 0.1	0.3 ± 0.1	0.3 ± 0.1	0.784
​ Glutamic oxaloacetic transaminase, IU/L	23.9 ± 6.1	23.2 ± 4.9	29.7 ± 11.7	20.3 ± 4.6	18.3 ± 3.8	23.7 ± 7.5	21.0 ± 4.8	19.2 ± 3.6	22.1 ± 3.7	0.256
​ Glutamic pyruvic transaminase, IU/L	33.7 ± 18.8	31.5 ± 15.5	32.8 ± 13.5	22.3 ± 13.3	20.3 ± 8.8	26.8 ± 14.0	20.6 ± 8.8	22.3 ± 7.9	25.0 ± 6.6	0.403
​ γ-glutamyl transpeptidase, IU/L	64.4 ± 58.4	49.1 ± 32.7	65.2 ± 68.1	42.7 ± 23.4	43.7 ± 27.0	53.8 ± 41.4	43.8 ± 55.2	40.1 ± 47.5	41.0 ± 43.3	0.094
​ Cholinesterase, IU/L	326.5 ± 39.0	342.1 ± 37.5	335.9 ± 28.9	363.8 ± 66.7	358.3 ± 67.5	360.8 ± 68.4	357.4 ± 55.6	363.3 ± 58.4	354.4 ± 59.6	0.273
​ NK cytotoxicity, %	31.6 ± 19.1	35.3 ± 18.3	27.1 ± 12.1	35.3 ± 17.6	37.6 ± 12.8	31.8 ± 10.6	35.2 ± 14.9	41.1 ± 13.9	36.1 ± 12.7	0.723
​ T cell, %	86.4 ± 6.5	88.6 ± 3.8	87.2 ± 4.0	88.8 ± 4.8	88.5 ± 5.0	87.3 ± 5.8	87.3 ± 4.0	87.6 ± 4.0	86.2 ± 4.1	0.425
​ B cell, %	6.7 ± 6.6	3.1 ± 1.9	5.9 ± 3.1	4.8 ± 4.3	3.1 ± 1.7	5.3 ± 3.2	4.3 ± 3.2	2.6 ± 1.2	5.3 ± 3.0	0.679
​ Cluster of differentiation 4+, %	40.7 ± 7.0	38.2 ± 5.5	40.2 ± 8.1	38.8 ± 9.4	37.6 ± 6.8	36.1 ± 5.0	43.7 ± 8.9	39.0 ± 9.9	37.7 ± 7.3	0.047
​ Cluster of differentiation 8+, %	36.7 ± 6.8	38.2 ± 6.8	36.4 ± 7.2	35.3 ± 10.3	35.9 ± 9.2	36.1 ± 8.1	33.0 ± 7.2	34.6 ± 6.4	35.8 ± 7.2	0.051
​ Cluster of differentiation 4/8	1.2 ± 0.4	1.0 ± 0.3	1.2 ± 0.5	1.2 ± 0.6	1.1 ± 0.4	1.1 ± 0.3	1.4 ± 0.7	1.2 ± 0.5	1.1 ± 0.5	0.031
​ Uric acid, mg/dL	6.7 ± 0.9	6.5 ± 1.0	6.6 ± 1.2	5.9 ± 0.9	5.9 ± 1.0	5.6 ± 1.0	5.9 ± 1.3	5.8 ± 1.1	5.5 ± 1.2	0.473
Mental state										
​ -Tension	52.3 ± 9.6	54.2 ± 6.9	53.5 ± 5.8	45.5 ± 7.6	47.8 ± 9.7	46.8 ± 8.4	45.8 ± 5.2	46.3 ± 8.1	47.1 ± 7.7	0.959
​ -Depression	55.6 ± 7.2	51.5 ± 5.9	53.6 ± 7.9	46.4 ± 5.3	48.9 ± 8.5	48.0 ± 6.7	46.9 ± 6.7	48.2 ± 8.7	47.8 ± 7.3	0.177
​ -Anger	52.6 ± 5.9	51.2 ± 6.0	52.2 ± 7.6	47.8 ± 9.1	48.8 ± 8.0	48.3 ± 6.4	44.4 ± 6.5	46.0 ± 8.3	44.7 ± 5.3	0.875
​ -Vigor	42.1 ± 5.6	46.9 ± 7.2	45.3 ± 5.0	44.2 ± 7.6	48.4 ± 10.4	45.6 ± 9.3	43.7 ± 11.3	43.0 ± 11.7	43.3 ± 10.6	0.229
​ -Fatigue	54.3 ± 11.8	54.7 ± 6.6	52.0 ± 8.1	48.3 ± 9.4	49.0 ± 9.2	49.4 ± 8.5	48.8 ± 8.9	50.6 ± 11.0	48.6 ± 10.4	0.893
​ -Confusion	58.4 ± 11.1	54.8 ± 13.1	55.4 ± 10.5	48.0 ± 6.0	49.8 ± 7.7	49.4 ± 7.3	51.0 ± 6.9	53.0 ± 8.9	52.0 ± 9.1	0.267

#: Variables not fulfilling Mauchly's test of sphericity were analyzed after Greenhouse-Geisser correction.

Table 6 shows the results of subgroup analysis of the process measures. A significant difference with the intervention group (*P* < 0.05) was observed only with smoking status.

**Table 6.** Subgroup analysis of lifestyle characteristics

Process Measurements		High adherence group (*n* = 10)			Low adherence group (*n* = 12)			Control group (*n* = 21)			*P*-value
		
		baseline	6 months later	follow-up	baseline	6 months later	follow-up	baseline	6 month later	follow-up	
Regular breakfast	yes	9 (90%)	8 (80%)	8 (80%)	10 (83%)	10 (83%)	10 (83%)	17 (81%)	18 (86%)	19 (91%)	0.212 0.153
	no	1 (10%)	2 (20%)	2 (20%)	2 (17%)	2 (17%)	2 (17%)	4 (19%)	3 (14%)	2 (10%)	
Number of sleep hours*	hours per day	7.3 ± 0.8	7.0 ± 0.8	7.1 ± 0.9	6.63 ± 0.9	6.89 ± 1.0	6.88 ± 0.9	6.86 ± 0.9	6.74 ± 0.8	6.81 ± 0.7	0.285
Snacking frequency	Seldom	5 (50%)	3 (30%)	3 (30%)	5 (42%)	5 (42%)	6 (50%)	8 (38%)	6 (29%)	10 (48%)	0.891 0.722
	1–2 times a month	0 (0%)	1 (10%)	1 (10%)	0 (0%)	1 (8%)	1 (8%)	1 (5%)	2 (10%)	1 (5%)	
	1–2 times a week	0 (0%)	1 (10%)	2 (20%)	3 (25%)	2 (17%)	4 (33%)	4 (19%)	3 (14%)	5 (24%)	
	3–4 times a week	3 (30%)	2 (20%)	2 (20%)	3 (25%)	1 (8%)	0 (0%)	2 (10%)	4 (19%)	4 (19%)	
	Almost every day	2 (20%)	3 (30%)	2 (20%)	1 (8%)	3 (25%)	1 (8%)	6 (29%)	6 (29%)	1 (5%)	
Drinking status	Drinker	10 (100%)	10 (100%)	7 (70%)	8 (67%)	10 (83%)	9 (75%)	16 (76%)	16 (76%)	16 (76%)	1.000 0.131
	Former drinker	0 (0%)	0 (0%)	0 (0%)	0 (0%)	0 (0%)	0 (0%)	0 (%)	0 (%)	0 (%)	
	Infrequent drinker	0 (0%)	0 (0%)	3 (30%)	4 (33%)	2 (17%)	3 (25%)	5 (24%)	5 (24%)	5 (24%)	
Amount of alcohol consumed*	Units^#^	1.59 ± 0.8	1.35 ± 0.6	1.36 ± 1.0	1.18 ± 0.8	1.06 ± 0.9	1.13 ± 1.0	1.03 ± 0.8	1.41 ± 1.1	1.03 ± 1.0	0.137
	(1 unit = 500 mL/day of beer, or equivalent)^#^										
Smoking status	Smoker	3 (30%)	3 (30%)	3 (30%)	5 (42%)	4 (33%)	3 (25%)	8 (38%)	7 (33%)	8 (38%)	0.612 0.017
	Former smoker	3 (30%)	3 (30%)	3 (30%)	3 (25%)	3 (25%)	3 (25%)	3 (14%)	5 (24%)	3 (14%)	
	Never-smoker	4 (40%)	4 (40%)	4 (40%)	4 (33%)	5 (42%)	6 (50%)	10 (48%)	9 (43%)	10 (48%)	
Daily stress	Very high	3 (30%)	4 (40%)	1 (10%)	2 (17%)	1 (8%)	1 (8%)	2 (10%)	3 (14%)	1 (5%)	0.684 0.596
	High	6 (60%)	3 (30%)	6 (60%)	3 (25%)	5 (42%)	5 (42%)	7 (33%)	7 (33%)	4 (19%)	
	Normal	1 (10%)	3 (30%)	2 (20%)	5 (42%)	3 (25%)	4 (33%)	9 (43%)	8 (38%)	15 (71%)	
	Low	0 (0%)	0 (0%)	1 (10%)	2 (17%)	3 (25%)	2 (17%)	3 (14%)	3 (14%)	1 (5%)	
Middle strength exercise*	min per month	391 ± 342.3	370 ± 420.2	212 ± 187.4	415 ± 495.6	323 ± 355.7	218 ± 367.7	162 ± 218.9	224 ± 298.2	320 ± 456.4	0.177
Awareness of the need for	Always aware	0 (0%)	1 (10%)	0 (0%)	0 (0%)	3 (25%)	0 (0%)	1 (5%)	1 (5%)	1 (5%)	0.568 0.190
physical activity	Usually aware	5 (50%)	3 (30%)	4 (40%)	8 (67%)	4 (33%)	9 (75%)	8 (38%)	8 (38%)	11 (52%)	
	Sometimes aware	2 (20%)	4 (40%)	5 (50%)	3 (25%)	4 (33%)	2 (17%)	6 (29%)	10 (48%)	7 (33%)	
	Seldom aware	3 (30%)	2 (20%)	1 (10%)	1 (8%)	1 (8%)	1 (8%)	6 (29%)	2 (10%)	2 (10%)	
Practice of	Precontemplation	0 (0%)	1 (10%)	1 (10%)	3 (25%)	0 (0%)	1 (8%)	5 (24%)	3 (14%)	6 (29%)	0.736 0.236
physical activities	Contemplation	4 (40%)	0 (0%)	2 (20%)	2 (17%)	4 (33%)	6 (50%)	4 (19%)	5 (24%)	7 (33%)	
	Preparation	2 (20%)	5 (50%)	2 (20%)	3 (25%)	6 (50%)	4 (33%)	5 (24%)	7 (33%)	4 (19%)	
	Action	2 (20%)	0 (0%)	0 (0%)	2 (17%)	0 (0%)	0 (0%)	2 (10%)	3 (14%)	1 (5%)	
	Maintenance	2 (20%)	4 (40%)	5 (50%)	2 (17%)	2 (17%)	1 (8%)	5 (24%)	3 (14%)	3 (14%)	
Frequency of spa bathing*	times per month	1 ± 1.2	2.3 ± 2.5	2.5 ± 3.0	1.83 ± 1.8	1.75 ± 2.4	1.5 ± 1.6	0.95 ± 1.9	1.29 ± 1.9	1.24 ± 2.6	0.191
Frequency of bathing at home*	times per month	23.2 ± 9.2	26.4 ± 2.5	24.3 ± 6.3	22.6 ± 8.4	22.5 ± 8.5	21.8 ± 9.5	23.9 ± 7.5	23.5 ± 6.6	24.1 ± 6.3	0.698

*: Continuous variables are shown as mean ± standard deviation and were tested by the paired *t*-test.

Categorical variables are shown as frequency (percentage).

The Kruskal-Wallis test was adapted to test the difference in 6-month change between groups (left *P*-value), it was also adapted to the change from baseline to follow-up (right *P*-value).

Rates of intervention attendance for the high and low attendance groups were 57.0 ± 22.3% and 8.3 ± 9.4%, respectively, and were significantly different (*P* < 0.001). Participants who performed their individualized programs attended the classroom more frequently.

There were no adverse events during the intervention or examination. There were also no cases of hospitalization or severe morbidity during the study period.

## DISCUSSION

In this study, the intervention was based on the working conditions of male white-collar employees, and was designed to be feasible for industrial health. The study was completed successfully: no dropouts were observed and a complete ITT analysis was performed. This allowed us to precisely evaluate the duration of the effects from the end of the intervention to 1 year after the intervention.

No significant effects were observed in the primary outcome measures, HDL-C. Also, a minor inclination toward beneficial activities was observed in the process measurements used to explain causal relationships. The ineffectiveness of the intervention might have been due to low attendance and poor adherence (56.2 ± 24.2% and 30.5 ± 29.6% in the intervention and observation periods, respectively). In addition, we did not attempt to forcefully persuade participants to engage in physical activity or to change their behavior, out of consideration for their job-related fatigue and the importance of time for their family life.

A recent study reported that variability in adherence to an unsupervised exercise regimen in obese women was poor^28^; an unsupervised individualized program was also employed in the present study. A systematic review^1^ showed that booster interventions using the telephone, e-mail, or the Internet strengthened the long-term duration of the effects, and an RCT^29^ showed that brief, monthly personal contact provided a modest benefit in sustaining weight loss. These findings suggest that distributing black-and-white leaflets only 4 times after the intervention was insufficient to augment the effects of the present intervention. In addition, only a small number of participants had specific diseases that necessitated immediate changes in their behavior, and the intervention was performed for the purpose of general health improvement. Therefore, the results might be partially due to insufficient participant receptiveness to the intervention.

Regarding the criteria used for immunological evaluation, CD4+ represented the role of helper T cells in relation to cytokine generation and macrophage activation. We found that high values of CD4+ were maintained in the intervention group, especially in the high adherence group, and that values tended to decrease in the control group. CD4/8, a secondary outcome measure, also showed some interaction. Participants who performed satisfactorily in the individualized programs had better class attendance. This suggests that those who had diligently learned techniques for improving their health from the specialist trainers—and who had good adherence to their self-constructed weekly additional physical activities and hot spa bathing—maintained and improved their immunological function for a period as long as 18 months. Improvements in health-related behavior, stress relief, and relaxation due to spa bathing^20^ may influence immunological function via automatic nervous system activity,^19^ but the mechanism underlying such an effect is unclear. In addition, participants in the high adherence group experienced a greater decrease in body fat percentage than did the other groups.

All the participants in this study were automobile commuters, as they resided in areas where the mass transportation system was less developed than in the major urban areas of Japan. This limited the physical activity in their daily lives. A nationwide population-based study in Australia supported recommendations to cycle to work or use public transport, rather than cars, as a strategy to maintain healthy weight in men.^30^ Moreover, a recent study reported that, among middle-aged white-collar office workers, full-time work was associated with lower rates of achieving recommended physical activity levels.^9^ The study also discussed techniques for increasing physical activity levels during automobile commuting and other times.

Recently, the concept of nonexercise activity thermogenesis (NEAT)^31^^,^^32^ has received attention as a way to increase energy consumption. This concept involves increasing the duration of physical activity by means of non-exercise activities, such as posture maintenance, walking for commuting or shopping, or load carrying during work. On the JALSPAQ, no significant difference was observed between the intervention and control groups with respect to moderate exercise. Considering that all the participants were automobile commuters living a sedentary lifestyle, an intervention that encouraged moderate physical activity might have been insufficient.

Studies have shown that adherence to healthy lifestyle behaviors is associated with a lower risk of acute coronary syndrome among obese individuals,^6^ and that current health and health-related behaviors were stronger predictors than social factors of early mortality among older women.^7^ These findings highlight the importance of comprehensive health education on factors such as diet, smoking cessation, and sleep hygiene, in addition to the importance of physical activity.

In the present study, hot spa bathing was employed as a unique intervention, and we recommended that participants bathe rather than shower at home. Despite the many studies on balneotherapy, evidence for its effectiveness is weak, due to difficulties in obtaining definite results and methodological problems with the RCTs that have been performed.^33^^–^^35^ However, hot spas are being increasingly utilized in many countries,^12^^–^^14^ with almost no adverse reactions or health hazards reported,^35^ and this has enhanced the utilization of hot spa bathing in health promotion campaigns. It was reported that, even after a 1-year observation period, improvements in HbA1c, aerobic capacity, back pain, vigor, fatigue, and depression were maintained in middle-aged women who had received comprehensive health education that included instruction on lifestyle and exercise, in combination with hot spa bathing once per week for 6 months.^36^ A cohort study reported that repeated comprehensive health education classes that included instruction for lifestyle, exercise, diet, and spa bathing, although available only biweekly, may have been effective preventive care for middle-aged and elderly people.^37^ Both the effectiveness and the cost-benefit of an intervention—especially as it relates to curbing medical expenses—are important concerns. To cite one example, a cohort study showed that medical expenses for subjects walking more than 1 hour per day were lower than for those walking less.^38^ The present report also emphasized the importance of maintaining a favorable lifestyle, from the perspective of medical economics. Unfortunately, the office in charge of health insurance for the participants was unable to provide information on individual medical expenses incurred during the study period.

Comprehensive intervention by means of health education once every 2 weeks for 24 weeks, as performed in this study, is feasible in many white-collar labor environments, although poor results should be expected. However, substantial effects might be observed with a group approach that used definite selection criteria for the participants (eg, improvements in obesity or lipid metabolism) and specified a target based upon such criteria. In addition, regular communication with participants might maintain their motivation.

This study had some potential limitations. First, although comprehensive health education was performed by specialists in a number of fields, improvements in diet were limited. Second, a type 2 error may have occurred in the subgroup analysis due to the small number (10 to 12) of subjects in each arm of the intervention groups. Third, because the participants were government employees of a single municipality, participants in both groups might have met one another and learned their group assignments. As a result, participants in the control group could have been unintentionally informed of the activities in the intervention group. The tendency observed in the control group—improvements in healthy behaviors (increased physical activity) and in physical measurements and some blood profile parameters—might not have been due exclusively to the Hawthorne effect, but could have also been influenced by such hearsay. This possibility may necessitate the use of a different study design, such as cluster RCT. Fourth, although it was a considerable achievement that there were no dropouts during this study, there were participants whose rates of attendance and adherence were very poor. We were unable to identify the reasons for these poor outcomes; thus, evidence-based measures could not be established in this study. Finally, we could not perform subgroup analysis between participants within and outside of reference values for each primary outcome measurement.

## CONCLUSION

Participants who attended classes and/or participated in additional individualized programs showed a tendency to maintain their immunological functions and to experience a decrease in body fat percentage. Lesser effects were observed in participants with poor adherence, even in the intervention group. However, we cannot quantify the effectiveness of each intervention (eg, spa bathing, exercise, healthy behavior, etc.) due to difficulties in interpretation that result from the inclusion of a comprehensive education program for the participants.
